# Drug Utilization Study in Ophthalmology Outpatients at a Tertiary Care Teaching Hospital

**DOI:** 10.1155/2013/768792

**Published:** 2013-12-22

**Authors:** Pradeep R. Jadhav, Vijay V. Moghe, Yeshwant A. Deshmukh

**Affiliations:** Department of Pharmacology, MGM Medical College & Hospital, Sector 18, Kamothe, Navi Mumbai, Maharashtra 410 209, India

## Abstract

In view of the advancement in drug development and availability of new ocular therapeutics in the discipline of ophthalmology, we attempted to study the drug utilization and describe the prescribing practices of ophthalmologists in a tertiary care teaching hospital. *Method*. A prospective, cross-sectional, observational study was conducted on patients attending Outpatient Department of Ophthalmology for curative complaints. Prescriptions of 600 patients treated were analyzed by the WHO prescribing indicators and additional indices. *Results*. Analysis showed that the average number of drugs per prescription was 1.49. Percentage of drugs prescribed by generic name was 2.35%. Percentage of encounters with antibiotics was 44.83%. Percentage of drugs prescribed from National Essential drug list (NEDL)/National Formulary of India (NFI) was 19.48%. Patient's knowledge of correct dosage was 93.83%. Antimicrobial agents were the most commonly prescribed drugs followed by antiallergy drugs and ocular lubricants. Fluoroquinolones accounted for 60% of the total antimicrobial drugs, of which gatifloxacin was the most frequently prescribed fluoroquinolone. *Conclusion*. The study indicated an awareness of polypharmacy, but showed ample scope for improvement in encouraging the ophthalmologists to prescribe by generic name and selection of essential drugs from NEDL/NFI.

## 1. Introduction

The World Health Organization (WHO) has defined drug utilization research as the marketing, distribution, prescription, and use of drugs in a society, with special emphasis on the resulting medical, social, and economic consequences [1–3]. It is an essential part of pharmacoepidemiology which describes the extent, nature, and determinants of drug exposure with the ultimate goal to facilitate rational use of drugs in the population [1–4].

Drug therapy is a major component of patient care management in health care settings. Prescribers and consumers are flooded with a vast array of pharmaceutical products with innumerable brand names, available often at an unaffordable cost [5]. Irrational and inappropriate use of drugs in health care system observed globally is a major concern [1, 6, 7]. To address the rising microbial resistance, physicians readily accept and indiscriminately use newly developed expensive and broad spectrum antibiotics which further contribute to increase rates of antimicrobial resistance and health care costs [8].

Recently in the discipline of ophthalmology, there have been many drug developments and introduction of new ocular therapeutic agents [9, 10]. Antibiotics are widely prescribed for various ophthalmic diseases. Evidences have shown trends of resistance to different class of antibiotics often used in ocular therapeutics [11–13]. Indiscriminate use of topical antibiotics and nonsteroidal anti-inflammatory drugs cause histological and structural changes in conjunctiva [14, 15]. In order to improve drugs therapeutic efficacy, minimize adverse effects, and delay development of resistance, drug utilization trends and patterns need to be evaluated periodically [2, 16]. Therefore, the present study was undertaken with the aim to investigate drug utilization and prescribing practices of ophthalmologists with emphasis on antimicrobial utilization in a tertiary care teaching hospital in Navi Mumbai.

## 2. Material and Methods

### 2.1. Study Design & Method

This was an open label, prospective, cross-sectional, observational study conducted in Outpatient Department (OPD) of Ophthalmology in a tertiary care teaching hospital in Navi Mumbai. Approval of Institutional Ethics Committee was obtained. The sample size was kept 600 in accordance with the World Health Organization manual [1]. The study was conducted for one year duration between March 2008 to March 2009 in which newly registered adult patients from either sex who visited the Ophthalmology Outpatient Department for curative complaints was included (cases of red eye, discharge from eyes, itching, redness foreign body sensation, swelling, foreign body, raised intraocular pressure, and eye trauma were included). Cases of refractive errors, cataract, postoperative followups, any diagnostic test/procedure, repeat attendance, and patients not willing to give informed consent were excluded from the study.

Prescriptions of 600 patients treated during the course of the study fulfilling the inclusion and exclusion criteria were audited prospectively using a specially designed case record form (CRF) to record the required information from the OPD prescription cards of each patient. The details of prescribed drugs were recorded, including its dosage form, route of administration, frequency of administration, indications, and duration of therapy. Patient's knowledge about the correct dosage was also assessed. The recorded data was then analyzed by the WHO/International Network for Rational Use of Drugs (INRUD) core drug use prescribing indicators [1] and additional indices.

### 2.2. Data Analysis

#### 2.2.1. WHO/INRUD Drug Use Indicators

Average number of drugs per encounter, percentage of drugs prescribed by generic name, percentage of encounters with antibiotics prescribed, percentage of encounters with an injection prescribed, percentage of drugs prescribed from the National Essential Drug List (NEDL)/National Formulary of India (NFI) [17, 18], and patients' knowledge of correct dosage were analyzed.

#### 2.2.2. Additional Indices

Commonest class/type of antimicrobial agent prescribed, percentage of antimicrobials prescribed from NEDL/NFI, and distribution of ophthalmic conditions treated with antimicrobials.

#### 2.2.3. Statistical Analysis

Descriptive statistics were performed. Data was entered and analyzed with Microsoft Excel 2003. Values were expressed as actual numbers, percentage, and mean.

## 3. Results

In this study six hundred prescriptions (*n* = 600) were analyzed and the total number of drug products prescribed were eight hundred and ninety three (893). During this study, the number of drugs per prescription varied from zero to four (Table 1) and the average number of drugs per prescription was 1.49. Drugs were prescribed in four different dosage forms. Eye drops were the most commonly prescribed (79.51%) 710 dosage form, followed by ointment (15.23%) 136, capsules (2.69%) 24, and tablets (2.57%) 23. Drug dosage, frequency, and duration of treatment record were mentioned in 96.16% (577/600), 96.16% (577/600), and 95.33% (572/600) of prescriptions, respectively.

Out of total prescribed drugs, 97.65% (872) drugs were prescribed by brand name and only 2.35% (21) by generic name. Prescribing by brand name dominated. Use of antibiotics was frequent and the percentage of encounters/cases with antibiotics was 44.83% (269/600). The percentage of drugs prescribed from NEDL/NFI was 19.48% (174/893) and patients knowledge of correct dosage for prescribed drugs was 93% (563/600 cases).  Table 2 summarizes WHO/INRUD drug use indicator values.

During the study period the commonly prescribed classes of drugs are depicted in Table 3. Antimicrobial agents constituted 43.11% (385/893) and were the most commonly prescribed drugs either as single antibiotic (213/385) or fixed-dose combination (FDC) of antibiotics (122/385), FDC-antibiotic combination with nonsteroidal anti-inflammatory drugs (43/385), and FDC-antibiotic combination with steroids (7/385). Antiallergy drugs were the second common drugs prescribed, while ocular lubricants were at third position.

**Figure 1:**
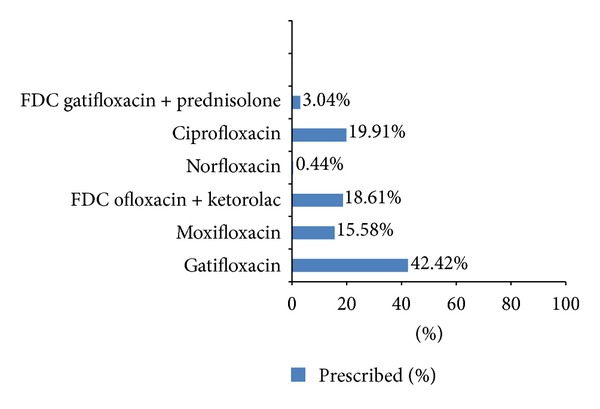
Different types of fluoroquinolones prescribed.

Fluoroquinolones were the most commonly prescribed antimicrobial class (Table 4). Fluoroquinolones accounted for 60% (231/385) of the total prescribed antimicrobials, of which gatifloxacin was the most frequently prescribed among the fluoroquinolones (Figure 1).

In this study, the antimicrobial agents were prescribed for infective conjunctivitis 16.10% (62), allergic conjunctivitis 14.02% (54), meibomian gland dysfunction 31.94% (123), stye 8.83% (34), blepharitis 12.72% (49), corneal abrasion-ulcer/keratitis 4.67% (18), foreign body removal 7.27% (28), uveitis 1.81% (7), and preseptal cellulitis 2.59% (10). Prescribing of antibiotics was rightly indicated except for allergic conjunctivitis, where we presume that antibiotics were prescribed for purulent discharge or secondary infection. Among the prescribed antimicrobial agents, only 34.54% (133/385) were prescribed from NEDL/NFI.

## 4. Discussion

Drug utilization studies are important for obtaining data about the patterns and quality of use, the determinants of drug use, and the outcomes of use. The WHO drug use indicators are highly standardized and are recommended for inclusion in drug utilization studies [1–3]. The present study attempts mainly to describe the current prescribing pattern and drug utilization with the WHO core prescribing indicators in Ophthalmology Outpatient Department.

Average number of drugs per prescription is an important index as it tends to measure the degree of polypharmacy [1]. It provides scope for review and educational intervention in prescribing practices. In this study the average number of drugs per prescription was 1.49, which demonstrated a restraint on over prescribing and polypharmacy to avoid risk of drug interactions. Other hospital-based studies in ophthalmology have reported higher value [19–24].

The percentage of drugs prescribed by generic name was 2.35% which was very low compared to other studies [19–24]. Most of the drugs were prescribed by brand name (97.65%) in this study, which suggests popularity of brands amongst the ophthalmologist and the influence of pharmaceutical companies. Ophthalmologist are reluctant to prescribe drugs by generic name presumably because it may result in the purchase of drugs of variable potency and underpotent generic antibiotics which may contribute to drug resistance and variability in clinical response [25]. However, prescribing drugs by generic name makes the treatment low cost and rational as it avoids prescription writing errors and confusion of dispensing of different brand names which sound alike and spell similar [23].

The percentage of drugs prescribed from the NEDL/NFI was 19.48% which is lower compared to studies conducted in India [5, 19]. This could be related to lack of awareness and unavailability of NEDL/NFI among ophthalmologist.

Antibiotics were frequent and number of encounters with antibiotics was 44.83%. Other hospital-based studies in ophthalmology in India have reported 14%–33% encounters with antibiotics lower than our study [19–23]. The high use of antibiotics may reflect the severity of infections and low sanitation in the region.

Patient's knowledge of correct drug dosage schedule was 93.83%. Patient's knowledge of correct dosage schedule ensures adherence to treatment compliance without indiscriminate use and promotes rational drug use.

Antibiotics constituted 43.11% (385) of the total drugs prescribed. Out of which 55.33% (213) were single antibiotics and 44.67% (172) were prescribed as fixed-dose combination (FDC) with other antibiotics, nonsteroidal anti-inflammatory drugs (NSAID), and glucocorticoids.

Fluoroquinolones were the most common group of antibiotics prescribed which were similar to reports of previous studies done in ophthalmology [19–23, 26]. Gatifloxacin was preferred in this study compared to other studies which documented ciprofloxacin [23, 26] and ofloxacin [22] as gatifloxacin was a new generation fluoroquinolone with a wider spectrum of activity against Gram negative as well Gram positive organisms, less side effects [9, 27, 28] and due to reports of emergence of resistance to other ocular antibiotics [11]. It was prescribed for various ocular conditions which included infective conjunctivitis, uveitis, stye, blepharitis, meibomian gland dysfunction, allergic conjunctivitis with purulent discharge, and preseptal cellulitis.

The percentage of antibiotics prescribed from NEDL/NFI was 34.54% which was also on the lower side and reflected lack of concept regarding essential drugs.

A rising trend in the prescribing of topical antiallergy (olopatadine and ketotifen) and ocular lubricants was documented in this study. This could be due to availability of emerging new efficacious drugs in the management of allergic conjunctivitis and dry eye syndrome [29, 30].

*Limitation of the Study*. It was a quantitative type of drug utilization study with the WHO/INRUD core prescribing indicators and therefore determining the quality of diagnosis and the appropriateness of drug choices was beyond the scope of prescribing indicators.

## 5. Conclusion

The prescribing pattern observed in the current study was knowledge-based and in accordance with the accepted patterns of treatment of ocular diseases, but the study showed ample scope for improvement in encouraging the ophthalmologists to prescribe by generic name and selection of essential drugs from NEDL/NFI. The study suggests educational initiative, development of drug policy, and NEDL-based hospital formulary to reduce the drug cost and ensure rational use of medicines.

## Figures and Tables

**Table 1:** Number of drugs prescribed per prescription.

Prescription containing number of drugs	Number of prescriptions (%)
Zero (none)	24 (4%)
One	289 (48.17%)
Two	262 (43.67%)
Three	20 (3.33%)
Four	5 (0.83%)

Total = 893	Total = 600 (100%)

**Table 2:** Details of drug utilization based on WHO/INRUD indicators.

Indicators assessed		Data value
1	Average number of drugs per encounter	1.49
2	Percentage of drugs prescribed by generic name	2.35%
3	Percentage of encounters with an antibiotic prescribed	44.83%
4	Percentage of encounters with an injection prescribed	0%
5	Percentage of drugs prescribed from national essential drug list/formulary	19.48%
6	Patients knowledge of correct dosage	93.83%

**Table 3:** Different types of drug products prescribed.

Sr. no.	Type	Number (Out of 893)	Percentage
1	Antibiotics	385	43.11%
2	Antiallergy	164	18.37%
3	Ocular Lubricants/artificial tears	137	15.34%
4	NSAIDs	90	10.08%
5	Ocular Decongestants	90	10.08%
6	Vitamins	12	1.34%
7	Mydriatics	11	1.23%
8	Antiglaucoma drugs	04	0.45%

**Table 4:** Prescribing frequency of antimicrobial drug classes.

Sr. no.	Antimicrobial Classes	Number (Out of 385)	Percentage
1	Fluoroquinolone	231	60%
2	Penicillin	05	1.3%
3	Tetracycline	23	5.9%
4	Chloramphenicol	59	15.3%
5	Macrolide	00	0%
6	Aminoglycoside	4	1.04%
7	Polypeptides and others	63	16.36%
